# Epidemiology of arrhythmogenic ventricular cardiomyopathy in China

**DOI:** 10.1002/clc.24160

**Published:** 2023-11-01

**Authors:** Si‐Tong Liu, Rui Li, Jian‐Peng Zheng, Feng Lu, Hui‐Ni Sun, Lin Hua, Gregory Y. H. Lip, Peng Zhong, Ying Bai

**Affiliations:** ^1^ Cardiovascular Center, Beijing Tongren Hospital Capital Medical University Beijing China; ^2^ Department of Pathology Beijing Tongren Hospital, Capital Medical University Beijing China; ^3^ Beijing Municipal Health Commission Information Center Beijing China; ^4^ School of Electronic and Information Engineering Beijing Jiaotong University Beijing China; ^5^ School of Biomedical Engineering Capital Medical University Beijing China; ^6^ Liverpool Centre for Cardiovascular Science University of Liverpool Liverpool UK; ^7^ Liverpool Heart & Chest Hospital Liverpool John Moores University Liverpool UK; ^8^ Department of Clinical Medicine Danish Center for Health Services Research, Aalborg University Aalborg Denmark; ^9^ Clinical Medical Laboratory Beijing Tongren Hospital, Capital Medical University Beijing China

**Keywords:** arrhythmogenic ventricular cardiomyopathy, Chinese population, epidemiology

## Abstract

**Background:**

Arrhythmogenic ventricular cardiomyopathy (AVC) is a common cause of ventricular arrhythmias and mortality, but limited data are available from large Asian cohorts. Our aim was to explore the current status of AVC and second, we examined the prevalence of ventricular tachycardia (VT), heart failure (HF) and mortality in patients with AVC in the Chinese population.

**Hypothesis:**

At present, some studies have reported that the incidence of AVC is on the rise, which may be due to the increasing number of diagnostic methods for AVC. However, there is no epidemiological data on AVC in the Chinese population, so we speculate that the incidence of AVC in the Chinese population is increasing.

**Methods and Results:**

We studied 15 888 adults from the Beijing Municipal Health Commission Information Center (BMHCIC) registry database in China from January 2010 to December 2020, and calculated the average annual percentage change (AAPC). Second, we determined the incidence of VT, HF and mortality in patients with AVC. Of the 10 318 men and 5570 women who were screened by cardiac magnetic resonance or examined by myocardial biopsy, there were a total of 256 newly diagnosed AVC patients (mean [SD]: 37.54[17.10]; 39.45% female). The incidence of AVC increased from 7.60 (3.12‐12.06) in 2010 to 19.62 (11.51‐27.75) per 1000 person‐years in 2020. Males had higher incidence of AVC than females. The AAPC for the rising incidence of AVC was 8.9 %. Males had similar VT prevalence (70.32% vs. 62.38%, *p* = 0.19) and mortality (1.94% vs. 1.98%, *p* = 0.98) but lower HF prevalence (42.58% vs. 60.40%, *p* = 0.006), when compared to females. Radiofrequency ablation (RFA) was more likely to be performed in males (*p* = 0.006).

**Conclusions:**

The rising trend in AVC incidence was evident, with two‐fold increase by 2020. Males with AVC had similar VT prevalence and mortality rate, but HF prevalence were lower than females, perhaps impacted by RFA use.

## Graphical abstract

We studied 15 888 adults from the Beijing Municipal Health Commission Information Center (BMHCIC) registry database in China from January 2010 to December 2020, there were a total of 256 newly diagnosed AVC patients. Males had higher incidence of AVC than females. Males had similar VT prevalence (70.32% vs. 62.38%) and mortality (1.94% vs. 1.98%) but lower HF prevalence (42.58% vs. 60.40%), when compared with females.

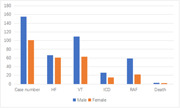

## 1 INTRODUCTION

Arrhythmogenic right ventricular cardiomyopathy/dysplasia (ARVC/D) is primarily genetically determined heart disease characterized by cardiac fibrofatty replacement of the right ventricle.^1^ Due to the increasing usage of imaging methods such as cardiac magnetic resonance imaging (cMRI), and improved genetic analyses in diagnosing ARVC, left ventricular fibrofatty replacement can be identified with a prevalence ranging from 10% to 50%, usually left‐dominant forms of ARVC.^2^, ^3^ AVC includes both kinds of diseases, with a higher risk of ventricular tachycardia (VT), heart failure (HF), and mortality.^4^

Pharmacotherapy, such as angiotensin converting enzyme inhibitors (ACEI) and β‐receptor antagonists, is the main treatment for AVC at present,^5^ and catheter ablation^6^ or implantable cardioverter defibrillator (ICD) is an option for VT treatment depending on the patient's estimated risk of sudden death, as well as their individual choice.^2^ If the disease is serious due to HF, cardiac resynchronization therapy‐defibrillator (CRT‐D) is considered. When a suitable donor is available, heart transplantation can be an option, but uptake is not high at present.

The prevalence of AVC varied widely depending on the studied population and the available diagnostic methods. For example, the prevalence ranges from 1/1000 in northern Italy to 1/5000–10 000 in Germany and other parts of the world.^7^, ^8^ However, few epidemiological studies are available on the incidence of AVC in the Chinese population.

We investigated the incidence of AVC in this retrospective study of 15 888 Chinese population investigated with at least one of the two protocols, which were cMRI and myocardial biopsy from 2010 to 2020. Second, we estimated the development of this disease with the trends estimation using annual percentage change (APC) and average annual percentage change (AAPC). Third, we explored the prevalence of VT (both sustained and nonsustained VT), HF, and mortality in the patients with AVC.

## 2 METHODS

### 2.1 Source of database

This registry study used the Beijing Municipal Health Commission Information Center (BMHCIC) database. BMHCIC is a mandatory health surveillance and supervision government agency requiring the medical information uploaded from all 153 hospitals/centers located in the overall Beijing area. The building of the data set was as previously described.^9^ The quality of the medical records was guaranteed through reviewing and analyzing the inspection results (https://www.phic.org. cn/).

The patients would receive ECG first and then they would be given echocardiography and angiography; also, signal‐averaged ECG, cMRI and myocardial biopsy were finally performed to ascertain the diagnosis of AVC. Their genes would be examined if the patients agreed. The registry covered the demographics information including sex, age, ethnicity, registered date, registered center, contact information, variation of diseases, examination methods, and vital status during each hospitalization and outpatient visits. The study protocol conformed to the ethical guidelines of the Declaration of Helsinki and was approved by the Ethical committee of our institution. Informed consent was waived due to anonymized and unidentified information for the analysis.

### 2.2 AVC and its complications (VT, HF, and mortality)

The diagnosis of AVC was established according to the 2010 diagnostic criteria^10^, ^11^ based on the patients' symptoms, signs, past history, and any clinical examinations, including 12‐lead electrocardiogram (ECG), 24‐hour Holter monitoring, echocardiography, angiography, signal‐averaged ECG, cMRI, myocardial biopsy, and treatment methods. The involvement of left and/or right ventricles were confirmed by at least one of the following examination methods, which were cMRI or myocardial biopsy. The final diagnosis was then stored in BMHCIC using diagnostic codes (International Classification of Diseases, Tenth Revision, Clinical Modification; ICD‐10‐CM) of I42.802 and/or I42.806. Only those with right ICD codes and at least one of cMRI or myocardial biopsy examinations were used for this analysis.

VT was diagnosed according to the established guidelines^12^ with diagnosis confirmed by 12‐lead ECG and 24‐hour Holter monitoring and stored using ICD‐10‐CM codes I47.201, I47.202, I47.203, I47.204, I47.205, I47.206, I47.207, I47.210, and I47.212. Due to the rapid progression of ventricular fibrillation in patients, it is difficult to record the results, so ventricular fibrillation was not considered in this analysis. Meanwhile, premature ventricular contractions were not included in this study because most normal people have premature ventricular contractions. HF was diagnosed according to established guidelines,^13^ with ICD‐10‐CM codes I 50. The first‐time diagnosis with AVC or AVC‐induced VT, and HF were considered as the date of onset for the condition. AVC, VT, and HF were assessed by independent cardiologists.

We selected the procedures of ICD implantment using codes 37.94002 or 37.94003; CRT with 00.51002; heart transplantation with 37.51001 and radiofrequency ablation (RFA) with 37.34001 and 37.34003 according to the International Classification of Diseases, Ninth Revision, Clinical Modification (ICD‐9‐CM).

### 2.3 Statistical analysis

The annual incidence of AVC was calculated with the number of newly diagnosed AVC cases divided by the population with cMRI or myocardial biopsy examinations in that referred year and expressed as per 1000 person‐year. Prevalences of VT, HF, and mortality were calculated by dividing the events using the corresponding AVC number in that referred year. The occurrence of incident AVC cases with year was assumed to conform to Poisson distribution and the time trends was evaluated with Joinpoint 4.9.0.0 (https://surveillance.cancer.gov/joinpoint/) with APC for each segment and AAPC for the whole trends.^14^ Joinpoint can be described as a turning point in a time series or trend, that is, a point of change from one trend to another, and can be used to describe the changing trend of some phenomena, such as the incidence, prevalence, and mortality of diseases, and to evaluate the effect of interventions and predict future trends. Incidence rate ratio (IRR) and 95% confidence interval (CI) of AVC cases with the potential effect of increased age and sex was calculated with the assumption of conforming to Poisson distribution. SPSS and Joinpoint software 4.9.0.0 were used for the calculation of the study. A *p* value < .05 was considered as statistically significant.

## 3 RESULTS

### 3.1 Incidence of AVC from 2010 to 2020

From 2010 to 2020, 15 888 patients had at least one of the two examinations which were cMRI or myocardial biopsy in the registry and of these, 921 had repeated examinations of cMRI or myocardial biopsy for confirmation of diagnosis.

Of these, a total of 256 newly diagnosed AVC were identified for the current analysis (mean age 37.54 [SD:17.10]years; 39.45% [*N* = 101] females). There were 3 athletes amongst them; 5 (1.95%) AVC were diagnosed after aborted SCD; 138 were diagnosed by cMRI; and 102 AVC were diagnosed by myocardial biopsy (16 patients were confirmed by both cMRI and biopsy).

Left ventricular dominated AVC were diagnosed in 32 (12.5%) patients, and both left and right ventricular involved AVC were diagnosed in 3 (1.17%) patients. Pathogenic mutation was seen in 89 of 125 patients with available genetic examination results, as shown in previous studies.^15^ The whole list of pathogenic mutations was unavailable for the current analysis. Baseline characteristics are shown in Table 1.

**Table 1:** Baseline characteristics of the diagnosed arrhythmogenic ventricular cardiomyopathy.

	AVC	ARVC	ALVC	Both ventricles involved
Number	256	221	32	3
Age	37.54 ± 17.10	36.87 ± 16.08	41.44 ± 22.29	45.00 ± 27.87
Female	101 (39.5)	84 (38.0)	14 (43.8)	3 (100.0)
Underlying diseases				
CHD	27 (10.5)	22 (10.0)	5 (15.6)	0 (0.0)
DM	17 (6.6)	14 (6.3)	3 (9.4)	0 (0.0)
Previous stroke	6 (2.3)	5 (2.3)	1 (3.1)	0 (0.0)
Complications				
New stroke	4 (1.6)	2 (0.9)	2 (6.3)	0 (0.0)
VTE	3 (1.2)	3 (1.4)	0 (0.0)	0 (0.0)
VT	172 (67.2)	152 (68.8)	18 (56.3)	2 (66.7)
HF	127 (49.6)	103 (46.6)	23 (71.9)	1 (33.3)
Treatment				
RFA	81 (31.6)	68 (30.8)	13 (40.6)	0 (0.0)
CRT‐D	2 (0.8)	1 (0.5)	1 (3.1)	0 (0.0)
ICD	41 (16.0)	34 (15.4)	6 (1.9)	1 (0.0)
Heart transplantation	51 (19.9)	50 (22.6)	0 (0.0)	1 (33.3)

Abbreviations: ALVC, arrhythmogenic left ventricular cardiomyopathy; ARVC, arrhythmogenic right ventricular cardiomyopathy; AVC, arrhythmogenic ventricular cardiomyopathy; CHD, coronary heart diseases; CRT‐D, cardiac resynchronization therapy‐defibrillator; DM, diabetes mellitus; HBP, hypertension; HF, heart failure; ICD, implantable cardioverter defibrillator; RFA, radiofrequency ablation; VT, ventricular tachycardia; VTE, venous thromboembolism.

**Figure 1:**
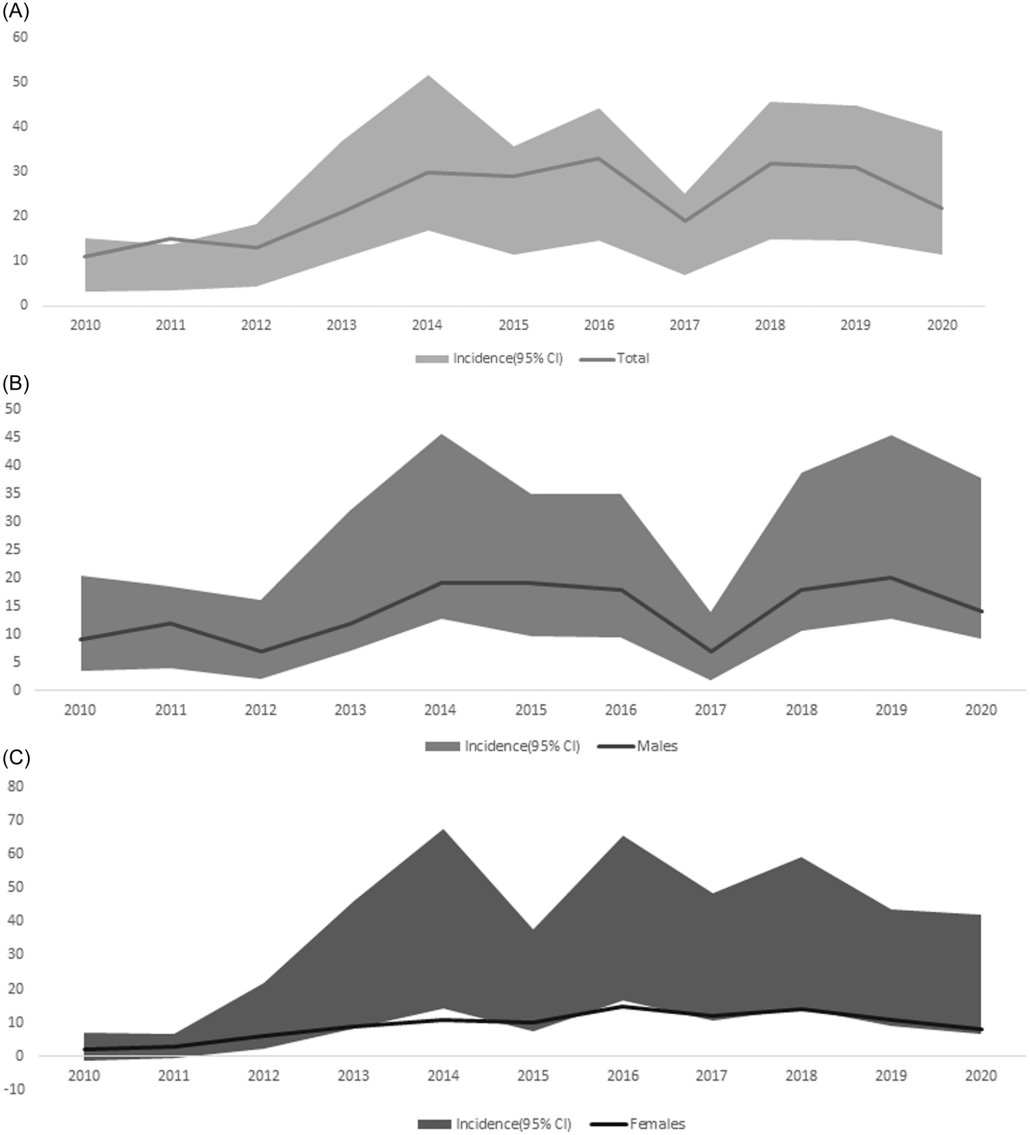
Incidence of arrhythmogenic ventricular cardiomyopathy. (A) Overall incidence of arrhythmogenic ventricular cardiomyopathy over time. (B) Incidence of arrhythmogenic ventricular cardiomyopathy in male over time. (C) Incidence of arrhythmogenic ventricular cardiomyopathy in female over time. AVC, arrhythmogenic ventricular cardiomyopathy.

**Figure 2:**
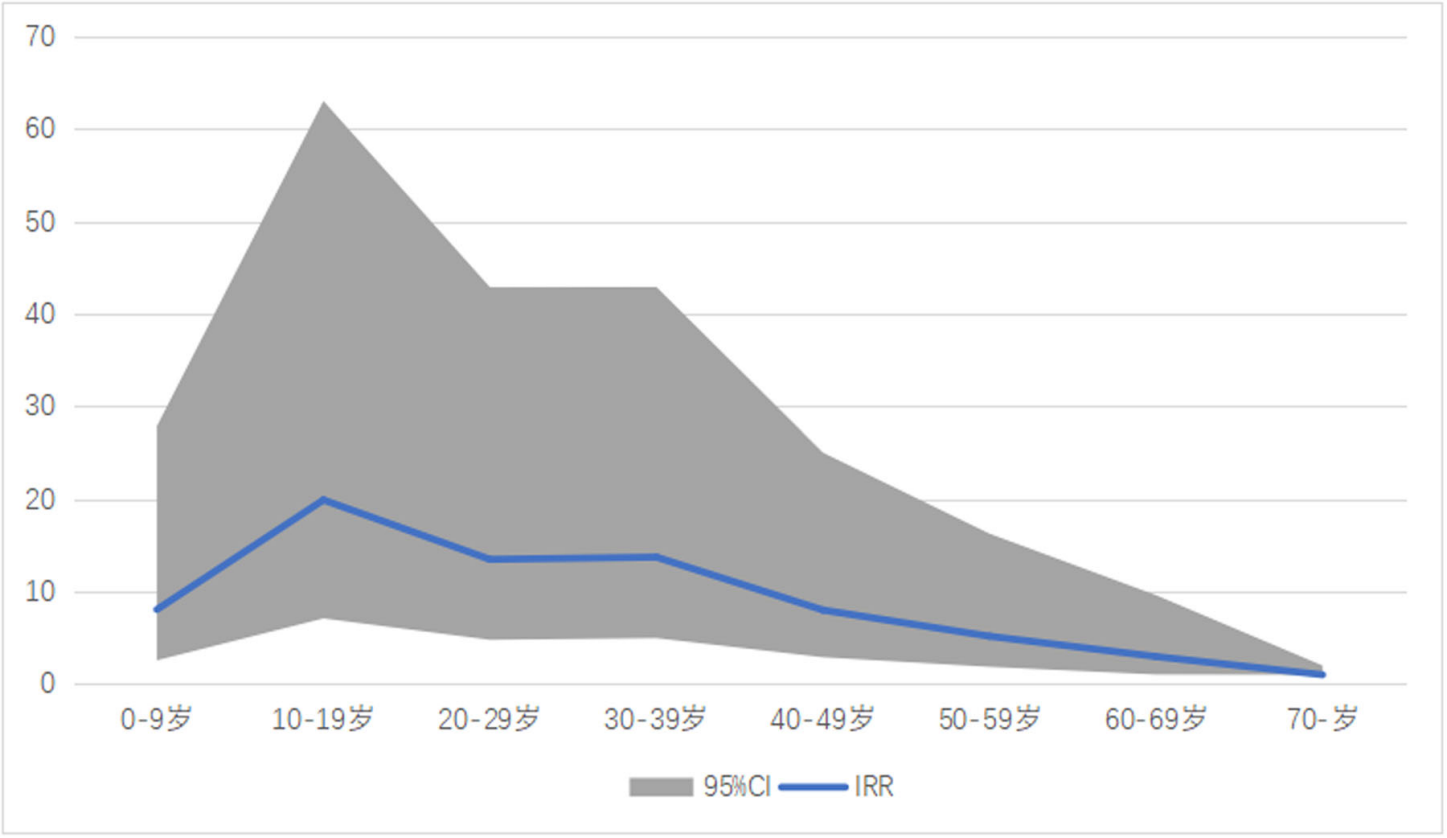
Incidence rate ratio (95% CI) of arrhythmogenic ventricular cardiomyopathy with ageing after adjustment of sex. AVC, arrhythmogenic ventricular cardiomyopathy.

The incidence increased from 7.60 (3.12–12.06) in 2010 to 19.62 (11.51–27.75) in 2020 per 1000 person‐years (Supporting Information: Table 1, Figure 1). The incidence of AVC maintained a high level at the age range of 10–39 years after adjustment of sex using Poisson distribution analysis as shown in Figure 2. The IRR of males to females was 0.74 (95% CI: 0.57–0.95) based on Poisson distribution analysis after adjustment of age.

### 3.2 Temporal trends from 2010 to 2020

As shown in Table 2, the increasing trends were seen in overall (AAPC = 8.9%, 95% CI: 0.3%–18.2%, *p* < .05), but not amongst males (AAPC = 7.0%, 95% CI: −0.7%–15.2%, *p* > .05) and females (AAPC = 10.3%, 95% CI: −3.1%–25.5%, *p* > .05) from 2010 to 2020. The APC increased sharply from 2010 to 2014 and was unchanged from 2014 to 2020 in females, albeit with stable trend in the two time periods (i.e., 2010–2014 and 2014–2020) in the males and sharply increased in the first period (i.e., 2010–2014) and was unchanged in the second period (i.e., 2014–2020) for the overall population. The AAPC increased to 12.7% (95% CI: 0.2%–26.9%, *p* < .05) and 27.2% (95% CI: 4.8%–54.3%, *p* < .05) for the total population and females respectively, when analyzed with 1 Joinpoint (All *p* < .05).

**Table 2:** Temporal trends of incidence of arrhythmogenic ventricular cardiomyopathy.

	Year range	Joinpoint	APC (95% CI)	*p* Value	AAPC (95% CI)	*p* Value
Total	2010–2014	1	37.9 (0.5–89.2)	*p* < .05		
	2014–2020	1	−1.4 (−13.1–11.9)	0.79		
	2010–2020	1			12.7 (0.2–26.9)	*p* < .05
	2010–2020	0			8.9 (0.3–18.2)	*p* < .05
Males	2010–2014	1	21.6 (−18.8–81.9)	0.16		
	2014–2020	1	0.8 (−14.8–19.3)	0.91		
	2010–2020	1			8.6 (−6.7–26.5)	*p* > .05
	2010–2020	0			7.0 (−0.7–15.2)	*p* > .05
Females	2010–2014	1	130.9 (8.2–392.5)	*p* < .05		
	2014–2020	1	−1.5 (‐12.2–10.5)	0.76		
	2010–2020	1			27.2 (4.8–54.3)	*p* < .05
	2010–2020	0			10.3 (−3.1–25.5)	*p* > .05

*Note*: Joinpoint 0 or 1 represents the number of turning point.

Abbreviations: AAPC, average annual percentage change; APC, Annual percentage change.

### 3.3 Prevalence of VT, HF, and mortality in AVC

Percentages of VT at first diagnosis was 67.19% (*N* = 172). Males had similar prevalence of VT to females (male vs. female: 109 [70.32%] vs. 63 [62.38%], *p* = .19). Of these patients, 41 underwent ICD implantation (male vs. female: 26 [16.78%] vs. 15 [14.85%], *p* = .68), and 81 received at least one RFA procedure for VT (male vs. female: 59 [38.06%] vs. 22 [21.78%], *p* = .006). The percentage with HF was 49.61% (*N* = 127) and males had lower prevalence of HF than females (male vs. female: 66 [42.58%] vs. 61 [60.40%], *p* = .005). In total, 51 heart transplantations were performed overall (male vs. female: 26 [16.77%] vs. 25 [24.75%], *p* = .12) and 2 CRT‐D implantations (male vs. female: 1 [0.65%] vs. 1 [0.99%], *p* = .98). Mortality rate was 1.95% (*N* = 5) of all the AVC patients, but this was lower in males than females (3 [1.94%] vs. 2 [1.98%], *p* = .98).

## 4 DISCUSSION

In this study, the incidence of AVC increased from 7.60 (3.12–12.06) in 2010 to 19.62 (11.51–27.75) per 1000 person‐year in 2020 with an AAPC of 6.7%. Second, AVC was associated with mortality rate of 1.95% and males had similar VT prevalence, mortality rate, and lower prevalence of HF when compared with females.

In the current analysis, AVC incidence in the current study was similar or higher than previously reported studies in Western countries.^7^, ^8^ We used information from the real‐world administrative data and studied 15 888 patients receiving at least one of the cMRI and myocardial biopsy for AVC diagnosis. Therefore, data of BMHCIC provides robust evidence for the epidemiological status of clinically relevant AVC in China. Therefore, this rising incidence of AVC could reflect the reality of AVC epidemiology in China.

Screening will be of great significance for prevention of sudden cardiac death, especially in those with probands. As for preventive measures, genetic testing has become an important diagnostic tool for screening patients because it is an inherited disease. Even in the absence of mutations, AVC is considered hereditary. Therefore, all family members should undergo diagnostic tests at intervals of 1–3 years.^5^ Due to the high VT prevalence and mortality rate preadmission,^8^, ^16^, ^17^ patients might not have chance to seek for medical care. As a result, facilitating access to medical care would be another most important factor contributing to prompt diagnosis and appropriate treatment of AVC.

To our knowledge, this is the first study revealing a rising trend of AVC incidence in the Chinese population, which could provide important information for formulating the prevention and treatment strategy. An increasing trend was indicated by the AAPC from 2010 to 2020, and such increasing trends were found both in males and females. This increasing trend was mainly seen in the age range from 10 to 39 years.

VT presented as main manifestations in a high proportion (67.19%), resulting in high risk of sudden cardiac death. Different from previous studies showing higher VT incidence in males,^18^, ^19^ males had similar prevalence of VT to females, but they had lower prevalence of HF and similar mortality rate in the current study. Surprisingly, RFA was more popular in males than females. Actually, RFA should be recommended for both men and women as an efficient preventive measure against HF and mortality.^15^ Exploring precise risk stratification also plays important role for those chose ICD or CRT‐D as prevention methods. Unfortunately, both of ICD and CRT‐D were not popular for treating AVC in China, perhaps due to fear of frequent burst caused by VT.

### 4.1 Limitations

There are some limitations for the current study. First, the clinical diagnostic data we used were mainly cMRI results or myocardial biopsy results, and only a few had detailed genetic test results, so there may have been some selection bias. Second, risk stratification could not be calculated due to lack of detailed information of probands and family members and their exercise burden. Third, the risk reduction management of drugs, such as beta‐blockers, antiarrhythmic drugs, and exercise reduction were unavailable for the current analysis. Fourth, few of the patients were diagnosed on postmortem given the low adoption of autopsy in the Chinese population. Fifth, patients who died of SCD before hospitalization were excluded from the current analysis which would cause an underestimation of AVC. Finally, the results of this study may be due, at least in part, to increased awareness of disease.

## 5 CONCLUSIONS

There was a rising trend in AVC incidence, with two‐fold increase by 2020. Males with AVC had similar VT prevalence and mortality rate, but HF prevalence were lower than females, perhaps impacted by RFA use.

## CONFLICT OF INTEREST STATEMENT

The authors declare no conflict of interest.

## Supporting information

Supporting information.

## Data Availability

We can provide relevant data if needed.
