# Supplementary material for: Epidemiology of arrhythmogenic ventricular cardiomyopathy in China
Source: Clin Cardiol. 2023 Nov 1;47(1):e24160. doi: 10.1002/clc.24160 (PMC10766133; doi:10.1002/clc.24160)
Supplement: Supplementary file 1 — Supporting information. [file CLC-47-e24160-s001.docx]

| **Supplementary Table 1** Incidence of arrhythmogenic ventricular cardiomyopathy | | | | | | | | | |
| --- | --- | --- | --- | --- | --- | --- | --- | --- | --- |
| Year | Total | | | Males | | | Females | | |
|  | New cases | cMRI number* | Incidence# | New cases | cMRI number* | Incidence# | New cases | cMRI number* | Incidence# |
|  |  |  | (95% CI) |  |  | (95% CI) |  |  | (95% CI) |
| 2010 | 11 | 1448 | 7.6(3.12,12.07) | 9 | 886 | 10.16(3.56,16.76) | 2 | 562 | 3.56(-1.36,8.48) |
| 2011 | 15 | 2203 | 6.81(3.37,10.24) | 12 | 1301 | 9.22(4.03,14.42) | 3 | 902 | 3.33(-0.43,7.08) |
| 2012 | 13 | 1411 | 9.21(4.23,14.2) | 7 | 865 | 8.09(2.12,14.06) | 6 | 546 | 10.99(2.24,19.73) |
| 2013 | 21 | 1141 | 18.4(10.61,26.2) | 12 | 749 | 16.02(7.03,25.01) | 9 | 392 | 22.96(8.13,37.79) |
| 2014 | 30 | 1158 | 25.91(16.76,35.06) | 19 | 832 | 22.84(12.69,32.99) | 11 | 326 | 33.74(14.14,53.34) |
| 2015 | 29 | 1620 | 17.9(11.44,24.36) | 19 | 1090 | 17.43(9.66,25.2) | 10 | 530 | 18.87(7.28,30.45) |
| 2016 | 33 | 1490 | 22.15(14.68,29.62) | 18 | 1031 | 17.46(9.46,25.45) | 15 | 459 | 32.68(16.41,48.95) |
| 2017 | 19 | 1508 | 12.6(6.97,18.23) | 7 | 1011 | 6.92(1.81,12.04) | 12 | 497 | 24.14(10.65,37.64) |
| 2018 | 32 | 1403 | 22.81(15,30.62) | 18 | 929 | 19.38(10.51,28.24) | 14 | 474 | 29.54(14.29,44.78) |
| 2019 | 31 | 1385 | 22.38(14.59,30.17) | 20 | 882 | 22.68(12.85,32.5) | 11 | 503 | 21.87(9.09,34.65) |
| 2020 | 22 | 1121 | 19.63(11.51,27.75) | 14 | 742 | 18.87(9.08,28.66) | 8 | 379 | 21.11(6.64,35.58) |

* Patients who received cardiac magnetic resonance and/or myocardial biopsy.

# number per 1 000 person-year.
